# Supplementary material for: Household serial interval of COVID-19 and the effect of Variant B.1.1.7: analyses from prospective community cohort study (Virus Watch)
Source: Wellcome Open Res. 2021 Dec 21;6:224. Originally published 2021 Sep 9. [Version 2] doi: 10.12688/wellcomeopenres.16974.2 (PMC8564743; doi:10.12688/wellcomeopenres.16974.2)
Supplement: Peer review history

# Peer review: Reviewer response for version 2

**DOI:** 10.21956/wellcomeopenres.19188.r47730

**Published:** 2021-12-22

**Authors:** Benjamin J. Cowling

**Affiliations:** 1 WHO Collaborating Centre for Infectious Disease Epidemiology and Control, School of Public Health, The University of Hong Kong, Hong Kong, China

No further comments.

Is the work clearly and accurately presented and does it cite the current literature?

Yes

If applicable, is the statistical analysis and its interpretation appropriate?

Yes

Are all the source data underlying the results available to ensure full reproducibility?

Yes

Is the study design appropriate and is the work technically sound?

Yes

Are the conclusions drawn adequately supported by the results?

Yes

Are sufficient details of methods and analysis provided to allow replication by others?

Yes

Reviewer Expertise:

Infectious disease epidemiology

I confirm that I have read this submission and believe that I have an appropriate level of expertise to confirm that it is of an acceptable scientific standard.

# Peer review: Reviewer response for version 2

**DOI:** 10.21956/wellcomeopenres.19188.r47729

**Published:** 2021-12-21

**Authors:** Vítor H. Nascimento

**Affiliations:** 1 Department of Electronic Systems Engineering, University of São Paulo, São Paulo, Brazil

I have no further comments on the paper.

Is the work clearly and accurately presented and does it cite the current literature?

Yes

If applicable, is the statistical analysis and its interpretation appropriate?

Yes

Are all the source data underlying the results available to ensure full reproducibility?

Partly

Is the study design appropriate and is the work technically sound?

Yes

Are the conclusions drawn adequately supported by the results?

Yes

Are sufficient details of methods and analysis provided to allow replication by others?

Yes

Reviewer Expertise:

Signal processing/statistics applied to epidemiology

I confirm that I have read this submission and believe that I have an appropriate level of expertise to confirm that it is of an acceptable scientific standard.

# Peer review: Reviewer response for version 1

**DOI:** 10.21956/wellcomeopenres.18741.r46856

**Published:** 2021-11-02

**Authors:** Benjamin J. Cowling

**Affiliations:** 1 WHO Collaborating Centre for Infectious Disease Epidemiology and Control, School of Public Health, The University of Hong Kong, Hong Kong, China

This is a valuable study of the household serial interval of COVID-19 based on the FluWatch cohort which includes tens of thousands of households from across the UK.

**Minor comments and suggestions:**

1. The title could be modified to "Household serial intervals ..." since the data reflect serial intervals in households but not other settings.
2. The exclusion of negative serial intervals might lead to overestimation of the mean serial interval? This could be clarified at the end of the second Discussion paragraph?
3. The standard deviation of serial intervals could also be presented in the abstract and also in Table 2 and the main text.

Is the work clearly and accurately presented and does it cite the current literature?

Yes

If applicable, is the statistical analysis and its interpretation appropriate?

Yes

Are all the source data underlying the results available to ensure full reproducibility?

Yes

Is the study design appropriate and is the work technically sound?

Yes

Are the conclusions drawn adequately supported by the results?

Yes

Are sufficient details of methods and analysis provided to allow replication by others?

Yes

Reviewer Expertise:

Infectious disease epidemiology

I confirm that I have read this submission and believe that I have an appropriate level of expertise to confirm that it is of an acceptable scientific standard.

# Peer review: Reviewer response for version 1

**DOI:** 10.21956/wellcomeopenres.18741.r45799

**Published:** 2021-09-27

**Authors:** Vítor H. Nascimento

**Affiliations:** 1 Department of Electronic Systems Engineering, University of São Paulo, São Paulo, Brazil

The paper compares the mean serial intervals for COVID-19 in areas with a large incidence of variant B.1.1.7, with areas in which the wild variant is dominant, concluding that there is no significant difference between the means.

 This comparison presents a number of difficulties, most of which are discussed in the paper. These difficulties are inherent to estimates of the serial interval distribution, and, although should be taken into account, in my opinion, do not invalidate the conclusions, since data about different variants was obtained at about the same time, using the same procedure.

- The data is restricted to infector-infectee pairs within households, so pairs with people in different households are not taken into account. This will probably bias down the observed serial intervals since contacts within households tend to be closer.
- In the introduction, it is mentioned that serial intervals and the doubling time of the epidemic are related to the reproduction number - but to be precise, it is the generation interval (time between infections) that has this relation. Since the generation interval is seldom observable, the serial interval is used as a proxy for it. I believe this should be acknowledged in the paper.
- Due to limitations in the software used, households with more than 6 people were not included in the study. It would be good to include in the text how many households are in this situation. One limitation of the study is that households with many people would tend to lower down the serial interval distribution, since if more people live together, they may presumably have closer contacts than in households with less people. However, since the same kind of bias was applied to all virus variants, I believe this would not affect the final conclusion.
- Asymptomatic cases were not included (since the serial interval is not defined for asymptomatic cases). This might be a source of bias if one variant has a larger percentage of asymptomatic infections than the other.  
- Negative serial intervals were also not considered (the absolute value of the serial interval is used instead). Previous studies report a rate of approximately 13% negative serial intervals (Griffin *et al.*, 2020).
- The distribution of serial intervals will depend on NPIs. Despite the fact that the study considers cases occurring in different regions at the same time, it might be important to check if different regions were not having different sets of restrictions in place (for example, in a region under lockdown, people would stay at home most of the time, potentially decreasing the within-household serial interval). This potential difference between hotspots was not discussed in the paper.
- Although the mean serial interval is particularly important, the whole distribution affects estimates of the reproduction number - so it might be worth comparing the distributions, not only their means.

The study is an important contribution to understanding the observed differences between variants, and a worthy contribution to the literature.

Is the work clearly and accurately presented and does it cite the current literature?

Yes

If applicable, is the statistical analysis and its interpretation appropriate?

Yes

Are all the source data underlying the results available to ensure full reproducibility?

Partly

Is the study design appropriate and is the work technically sound?

Yes

Are the conclusions drawn adequately supported by the results?

Yes

Are sufficient details of methods and analysis provided to allow replication by others?

Yes

Reviewer Expertise:

Signal processing/statistics applied to epidemiology

I confirm that I have read this submission and believe that I have an appropriate level of expertise to confirm that it is of an acceptable scientific standard.

# Response

**Published:** 2021-10-29

**Authors:** Cyril Geismar

**Affiliations:** University College London, UK

**1.The data is restricted to infector-infectee pairs within households, so pairs with people in different households are not taken into account. This will probably bias down the observed serial intervals since contacts within households tend to be closer.**

- We thank the reviewer for their valuable insights. We agree that the clustering method we implemented may impact our results. We state this limitation in the first paragraph of the Discussion section. To consider transmissions occurring outside the household we would require data about 1) the contact between members in our cohort and 2) data from those outside our cohort. Due to ethical and security concerns, point 1 is not possible as it requires us to have access to a deeper volume of data akin to surveillance for which we do not have ethical approval for. Point 2 is not possible as our study actively requires participants to knowingly provide data; we only have this at the household level.  

**2.** **In the introduction, it is mentioned that serial intervals and the doubling time of the epidemic are related to the reproduction number - but to be precise, it is the generation interval (time between infections) that has this relation. Since the generation interval is seldom observable, the serial interval is used as a proxy for it. I believe this should be acknowledged in the paper.**

- ​​​​​​​We thank the reviewer for pointing this out and will update for all audiences.

**3. Due to limitations in the software used, households with more than 6 people were not included in the study. It would be good to include in the text how many households are in this situation. One limitation of the study is that households with many people would tend to lower down the serial interval distribution, since if more people live together, they may presumably have closer contacts than in households with less people. However, since the same kind of bias was applied to all virus variants, I believe this would not affect the final conclusion.**

- We thank the reviewer for providing their insights into the limitation on household size. We agree that the bias applies to all virus variants and is not likely to impact the conclusion. Furthermore, according to the ONS report “Families and households in the UK: 2020”: “ *The average household size in the UK is 2.4 while there were 162,900 (0.6%) households in the UK with seven or more people*''. Therefore we believe that our results are representative. https://www.ons.gov.uk/peoplepopulationandcommunity/birthsdeathsandmarriages/families/bulletins/familiesandhouseholds/2020#:~:text=The%20average%20household%20size%20in,1.1%25%20in%20the%20West%20Midlands.
** **

**4. Asymptomatic cases were not included (since the serial interval is not defined for asymptomatic cases). This might be a source of bias if one variant has a larger percentage of asymptomatic infections than the other.  **

- We agree with the reviewer. We acknowledged in the paper that we were unable to measure asymptomatic transmissions and that the estimates provided are strictly measures of the serial interval when both the infector and infectee are symptomatic. 

**5. Negative serial intervals were also not considered (the absolute value of the serial interval is used instead). Previous studies report a rate of approximately 13% negative serial intervals (Griffin et al., 2020).**

- We thank the reviewer for pointing this out with supporting evidence from Griffin et al. (2020)’s rapid review. We did acknowledge the possibility of COVID-19 negative serial interval and explained why we were not able to consider this:
  “Although negative serial intervals are possible, in practice it is not possible to assess the direction of transmission between pairs, so it was assumed that the minimum interval was zero days.”
  Given the nature of our data (self-reported), we were not able to determine cases when transmission occurs prior to symptom onset.
  Although we acknowledge the possibility of a significant proportion of negative serial interval in COVID-19 transmissions, we do not believe that there is sufficient evidence from enough studies with significant transmission pairs to report a specific percentage.

**6. The distribution of serial intervals will depend on NPIs. Despite the fact that the study considers cases occurring in different regions at the same time, it might be important to check if different regions were not having different sets of restrictions in place (for example, in a region under lockdown, people would stay at home most of the time, potentially decreasing the within-household serial interval). This potential difference between hotspots was not discussed in the paper.**

- We thank the reviewer for their comments. To mitigate any variation related to the localised implementation of policies, our recruitment process was conducted using the royal mail list to recruit evenly across the UK; therefore our results are a close estimate to the average  and were less likely impacted by local variation.

** **


**7.Although the mean serial interval is particularly important, the whole distribution affects estimates of the reproduction number - so it might be worth comparing the distributions, not only their means.**

- We used the Welch two sample t test to compare VOC and non-VOC hotspots. This statistical test is a test of the distribution and not the mean itself as it accounts for the standard deviation which is a measure of the distribution. 
